# Introductory Lecture to the Course in Atlanta Medical College, for the Sessions of 1878-79

**Published:** 1878-10

**Authors:** J. T. Banks


ATLANTA
Medical and Surgical Journal.

Vol. XVI.] OCTOBER—1878. [No. 7

### Original Communications.

### INTRODUCTORY LECTURE
### TO THE COURSE IN ATLANTA MEDICAL COLLEGE FOR THE SESSIONS OF 1878-9, DELIVERED OCT. 15, 1878.

By Prof. J. T. BANKS.

Gentlemen: In behalf of my colleagues, I welcome you as students in the Atlanta Medical College.

This duty is made more pleasant by seeing so many familiar faces before me. Nothing can be more encouraging to us as a faculty. It gives confidence; it impresses on our minds the conviction that our past labors have been appreciated by you, and at the same time inspires a greater zeal in our renewed work. I bespeak for my colleagues a zealous and interested course of lectures—interested in the advancement you make in your studies, and interested in your success. The relationship which begins to-day between us as teachers and pupils I earnestly hope will be both pleasant and profitable. We would not have you feel as strangers toward us, for I assure you the same feeling and anxiety which you all experience as pupils, we have, under like circumstances, more or less felt; and a detailed history of medical students would fill a volume with repetition of similar feelings and emotions. While we command your confidence and respect, we will treat you collectively and individually as gentlemen. It may be well in this connection to call your attention to, and caution you against, an error too often committed. Think not that, because you are away from home—beyond the watchful care of parents and friends—medical students among strangers—that you can waste your hours for study in idleness, intemperance or improper company without cognizance of your deportment. At the very moment when you think no one heeds you, many eyes may be upon you. In your student's diary is noted your future character—your chances for success as doctors and as business men in the world. Then how important the record you make! In making your choice of a business for life, you have no doubt noted the vast amount of study, labor and self-denial that are necessary in your student life to insure professional success, and it is well calculated to discourage the beginner; but let me assure you, my young friends, that there is no profession worthy of being pursued which does not require much exertion, much labor and many sacrifices by those engaged in it; nor do I know of any in which he who has the necessary qualifications is surer of success. To succeed, you must have a fixed purpose. A thorough determination to attain an object is the first step toward its attainment. There must be no faltering, no turning to the right or left. Come to the lectures promptly and give them attention, and you shall be guided safely to the goal of your ambition. You have determined on your course, and are to-day in a great measure relieved of your responsibility to others, and in like manner are responsible to yourselves for your improvement of the passing hour. Your fortunes are in your own hands. You can make them or mar them, as you will. Fortuitous circumstances may do much to make the man; they may do more to undo the unqualified doctor. Grant that now and then the idle student is forced by his surroundings to cultivate his talents, and he grows into a practical and useful man. The road he travels is a rough one, and the number of such successes will only make the exceptions to a rule. Friends and fortune may place on the unmerited brow a dazzling crown, and it may be worn gracefully for an idle while—yea, it may be envied by the toiling student; it would be but natural. But on a time the hand of affliction will be extended for aid—the rushing life-blood from some injured artery must be arrested by the prompt application of the ligature. Skill, knowledge and confidence are required—decisive, wisely-directed action can alone save the sufferer. The tale is soon told in the death of one and ruin and disgrace of the other.

If your ambition is low, and you wanting in principle, your success will be mean and your position debased. If your ambition is high and principles just and noble, your success will be great and your position honorable.

Gentlemen, I would beseech you not to limit your present ambition to a successful examination at the close of the lectures. If that should be the limit of your ambition, it is easily obtained and you easily satisfied. A higher aim should be made, and much more is expected of you. Be not content with barely getting inside the green room dead-line. There is no good reason why a young man should not succeed in getting a diploma, if he has sense enough to entitle him to it.

Let us consider some of the causes of failure with medical students.

In the trivial affairs of life we often find the character of men written. "Save no dimes, and there will be no dollars to look after." He who travels a road listless and watchless can give no description of it. You cannot profit by hearing the lectures unless you study them. We see, then, that a careless inattention may be a prime factor in failure. Intemperance leads to bad results through the same channel, and at the same time impairs man intellectually and debases him morally. Too much reliance on books, to the neglect of didactic teaching and clinical observation, I have known to lead to a student's failure. It is bad to know too much and heed too little. Be not over-confident in your own learning, that your time may not be consumed in unlearning. We see, then, that an over-weening confidence on memory, without fixing the principles by study and comparison, may be a factor in failure. A life of pleasure is incompatible with a life of business. If the mind is taken possession of by a special vice, or a fondness for a favorite pleasure, it is made incapable to comprehend the sciences upon which the practice of medicine is founded, and failure must result. Too much thought and anxiety on a favorite subject creates a morbid sensibility, enervation of mind and dwarfing of intellect. The mind, like the body, is improved and strengthened by discipline and cultivation, but you must not forget that there is a limit to its capacity aud endurance. More than once have I seen bright minds ruined and driven to failures by the spur of ambition unwisely applied. It is true this is an error which occurs less often than its opposite, for man is naturally disposed to seek his ease and comfort, to shun danger and avoid responsibility. Your pride and ambition must urge you on, not only to success, but by honorable rivalry, to greatness, to fame and to honor. "If you would know what your own powers are, you must use them." All of you have talents—some more in one order, others more in another; let none of them be blighted by indolence, nor wasted in improper pursuits.

The practice of medicine is in a great measure an art—the successful application of which depends upon the knowledge of the several sciences pertaining to it—such as Physiology, Chemistry, Materia Medica, Anatomy and Pathology. To ignore these, or consider them of minor importance, is to sue for a seat on the lowest round, if indeed, any seat be attainable. Of these several sciences, you cannot know too much. The more thoroughly you acquaint yourselves with them, the easier your progress in everything pertaining to the profession.

The world expects to find in the Doctor a well-educated gentleman. There should be no disappointment. Society demands it; the profession demands it, and your own interest demands it. "There is no excuse for a young professional man, who does not devote some portion of his time to the general cultivation of his mind." All general knowledge, whether of literature, of moral or physical science, tends to expand the intellect, and qualify it better for particular pursuits. While we desire the highest possible degree of professional attainment, we cannot ask it through the entire disregard of such mental culture as society expects of Doctors. You can, in a great measure, select your own position in society: and let me assure you that if you are satisfied with a low or common one, you will not be given a high one. Do not underestimate your abilities—this error, when it exists, is about as pernicious as its opposite. Be not discouraged because you cannot progress as fast as you desire, or as fast as you may suppose some more favorably situated students. The best beginnings are not always the best endings. You cannot estimate yourselves by your present efforts; neither can your friends predict your future for you. The great men in medicine are not born geniuses, nor are they the off-spring of hasty, careless, or desultory efforts. They are the sons of hard, earnest, methodical work. "Work that will stand the test of time, and the criticism of competent judges." The midnight lamp has illuminated their pathway; a fixedness of purpose has guided their footsteps. They are "the evidences of trained thought and well-drilled intelligence." They made no haste to jump through the curriculum of study into the active work-shop of the Doctor—bankrupts in education and observation, and untrained in reasoning and thinking for themselves.

Here let me add the advice of Lord Stanley: "Study as long as in prudence you can, and don't fear that life will not be long enough to gather what you have sown." I assure you, gentlemen, the time you spend in well-directed study, will ever be looked back to with pleasurable satisfaction. To be independent, is a laudable ambition, and to make yourselves independent, you must first be useful. Prepare yourselves for difficulties—meet them in a proper spirit, make the necessary exertion for them. Be prompt and true to your engagements, both trivial and great. Discharge your duties faithfully, humanely and with dignity, and your success and independence are a certainty. You should be under no more obligations to those who employ you professionally than the landlord is to his tenant, or the banker to the man to whom he lends money.

The good feeling and confidence of your friends should be appreciated, and treated with proper consideration. To obtain a proper competency from your services for yourselves and families, is one of your prime duties; a consideration due your station in life, your comfort and convenience, and due your profession. "He that provides not for his own household, is worse than an infidel." It is, and has been, a noted fact, that our profession is too indifferent to its financial interest. No occupation or profession more justly merits a liberal compensation for their services than the medical, and it is a duty we owe to ourselves and our profession, to force the people to a just consideration of our claims. It is true, that humanity forbids the with-holding of our services from the truly needy, nor would I advise anything less than prompt, kind and faithful attention to them; but an intelligent public should by reason of our charity, be prompt and liberal toward us. But I am sorry to say, it is not as it should be. A great portion of the error is our own fault. If you fail to properly estimate your services, there is no way for correction, and indifference and excess of leniency becomes a ruinous fault. When pain and suffering forces open the purse-strings of the close or mean, take a just amount of "*change*," and it will not be regretted. They may be grateful and just in sickness and pain. They are sure to be forgetful, and may be grumblers in health. Be honest, faithful and just in all your actions, and demand prompt and reasonable compensation in return. Indulgence is rarely rewarded, though credit be a necessity, and friendship thus purchased, is sure to falter on pay-day. To be honest, faithful and just, is also essential for good character, and a good character is a large portion of your fortune. A good professional character, a good social character, and a good moral character, all must be acquired, and all sustained. Though your qualifications be perfect, without a good moral character you should not be admitted into the sick room. Show me a Doctor loose in morals, and unguided by the principles of right and justice, though he be a shining light and unsurpassed in business, I will show you in the end a miserable failure, bankrupted in character, and no chance to homestead—confidence lost, and no reclamation.

Your financial success should never be paramount to a good name. The one may furnish the wants and comforts of the hour, the other a heritage you have no right to barter—but keep, use and transmit. Never seek success at the cost of principle or by false promises, or you will find yourselves in the company of charlatans and quacks, without the good opinion and confidence of your community. Success gained by false pretension cannot last. Such usually end their days in disreputable want. The honor and prosperity of our noble profession will in a short time be entrusted to your care. Be sure of a good preparation for its keeping and its advancement. Give us evidence by your study and progress that you are determined to uphold it, learn to love and practice the golden rule of our profession. "Do unto others as you would have others do unto you," and you will become what you merit, and act as you will be—Professional Gentlemen.

